# The new Bruton’s tyrosine kinase inhibitors SPA8007 and SPA8009 reduce stemness and invasiveness of patient-derived glioblastoma tumorspheres

**DOI:** 10.1016/j.tranon.2025.102585

**Published:** 2025-10-26

**Authors:** Euna Jo, Eun Lee, Yoojung Oh, Dongkyu Lee, Byungho Lee, Kibyeong Kim, Ran Joo Choi, Jiyun Hong, Yuesong Jeon, Hyewon Cho, Yong-Sung Choi, Sangwoo Kim, So Young Won, Seonah Choi, Tae Hoon Roh, Ju Hyung Moon, Eui Hyun Kim, Jong Hee Chang, Raok Jeon, Seok-Gu Kang

**Affiliations:** a Department of Neurosurgery, Brain Tumor Center, Severance Hospital, Yonsei University College of Medicine, Seoul, 03722, Republic of Korea; b Brain Tumor Translational Research Laboratory, Department of Biomedical Sciences, Yonsei University College of Medicine, Seoul, 03722, Republic of Korea; c Department of Neurosurgery, Graduate School of Medical Science, Brain Korea 21 Project, Yonsei University College of Medicine, Seoul, 03722, Republic of Korea; d Brain Research Institute, Yonsei University College of Medicine, Seoul 03722, Republic of Korea; e College of Pharmacy, Sookmyung Women’s University, Seoul, 04310, Republic of Korea; f Department of Biomedical Systems Informatics, Brain Korea 21 PLUS Project for Medical Science, Yonsei University College of Medicine, Seoul, 03722, Republic of Korea

**Keywords:** Bruton’s Tyrosine Kinase inhibitor, Glioblastoma, SPA8007, SPA8009, Tumorsphere

## Abstract

Glioblastoma (GBM), the most prevalent primary brain tumor, remains incurable due to the presence of cancer stem cells (CSCs), which can be isolated as tumorspheres (TSs) that exhibit classical characteristics of CSCs, including stemness and invasiveness. The significantly elevated expression of Bruton’s tyrosine kinase (BTK) in GBM tissues identifies BTK as a potential therapeutic target in GBM. Consequently, ibrutinib, an FDA-approved BTK inhibitor for hematological malignancies, has been repurposed as a candidate for GBM treatment, demonstrating inhibitory effects on the proliferation, stemness, and invasiveness of GBM cell lines and TSs. However, its broad-spectrum activity targeting other Tec family kinases and members of the epidermal growth factor receptor family, poses potential life-threatening risks, necessitating the development of more selective alternatives. To address this shortcoming, we synthesized BTK-selective analogs (SPA1758, SPA1763, SPA8004, SPA8007, and SPA8009) specifically designed to target two hallmark features of GBM TSs: stemness and invasiveness.

WST and ATP assays identified SPA8007 and SPA8009 as the most effective candidates with superior cytotoxic effects in TSs. Additionally, both SPA8007 and SPA8009 significantly inhibited neurosphere formation and reduced invasiveness in GBM TSs. Furthermore, SPA8007 demonstrated improved survival rates in a GBM xenograft mouse model and significantly reduced the expression of invasiveness markers, as observed in immunohistochemistry analysis. These findings highlight SPA8007 as a potential novel chemotherapeutic agent with high specificity and efficacy to enhance GBM therapy.

## Highlights

• BTK is significantly elevated in patient derived GBM tissues and TSs.
• SPA8007 and SPA8009 suppress proliferation, stemness and invasiveness of GBM TSs.
• SPA8007 significantly improves survival in an orthotopic mouse xenograft model.
• SPA8007 is a potential novel chemotherapeutic agent in high BTK-expressing GBM patients.

## Graphical abstract

**Figure:**
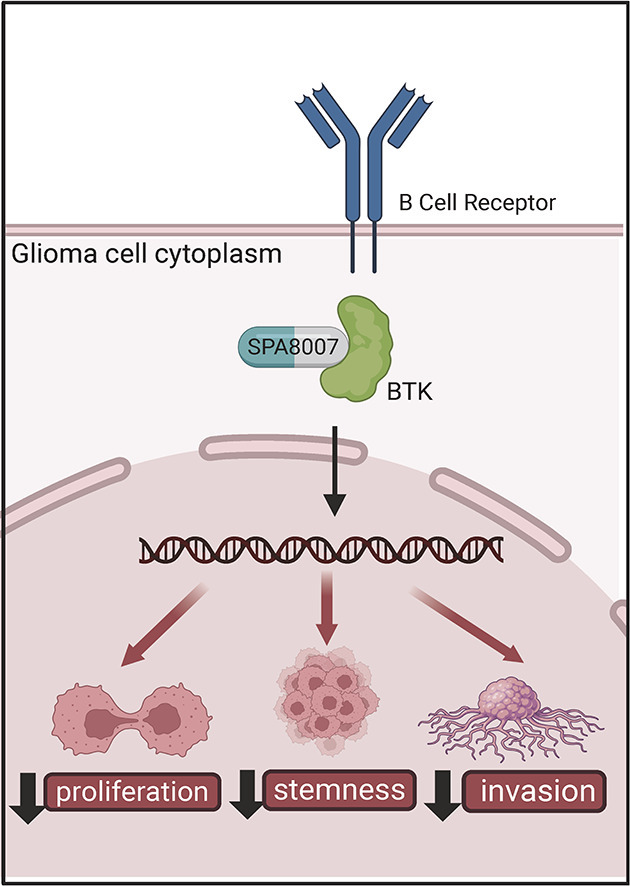

## Introduction

Glioblastoma (GBM) is the most aggressive and lethal primary brain tumor and accounts for 80 % of all central nervous system malignancies [11,15]. Despite treatment advances, the median survival remains low, at 15 to 20 months, due to the presence of cancer stem cells (CSCs) within the tumor, which drive tumor growth and treatment resistance [22,32,38,39,47]. Patient-derived GBM tumorspheres (TSs), representing CSCs, can be isolated and cultured from mother tissue, which presents phenotypic features such as stemness and invasiveness [18,37].

Studies have shown that elevated expression of genes associated with cancer aggressiveness and stemness is linked to high Bruton’s tyrosine kinase (BTK) expression in solid tumors [10,12,23,44,50]. Additionally, in GBM, high BTK levels are associated with poor prognosis, making BTK a promising therapeutic target [33,53]. BTK, a member of the large Tec family of non-receptor tyrosine kinases, plays a critical role in development, differentiation, and signaling pathways, extending its involvement into oncogenic pathways [34,35].

Ibrutinib, a prototype BTK inhibitor and US Food and Drug Administration-approved drug for hematological malignancy, has shown remarkable efficacy in reducing the proliferation, stemness, and invasion of GBM cells *in vitro* and *in vivo*, leading to over 400 registered clinical trials [9,43,46,55]. However, ibrutinib's broad spectrum of action targeting other Tec family kinases and members of the EGFR family causes serious side effects, including bleeding, hypertension, and atrial fibrillation, leading to therapy discontinuation in up to 32 % of clinical trials [6,40,54].

To overcome this drawback, we previously synthesized a set of five selective BTK inhibitors in-house by modifying lipophilic groups in the ibrutinib structure [24]. Kinase selectivity profiles of these compounds exhibited BTK selectivity greater than 92 %, with minimal selectivity toward other kinases such as ERBB2, EGFRK, ITK, JAK3, TEC, and TXK [2,24]. Furthermore, these compounds demonstrated *in vivo* efficacy in murine models of hematological malignancies, with no significant toxicity, as evidenced by clinical and histological assessments [2,24].

Given the potential of BTK inhibitors as effective TS targeting drugs, we hypothesized that they significantly reduce proliferation, stemness, and invasiveness in patient-derived GBM TSs. Indeed, our results confirmed the BTK inhibitors markedly reduced these traits both *in vitro* and *in vivo*, positioning these drugs as a leading candidate for GBM treatment.

## Materials and methods

### Sample isolation and culture

A total of 99 tumor tissues were collected from patients who underwent surgical resection at Severance Hospital, Yonsei University College of Medicine, Seoul, Republic of Korea. Tumor-free cortex samples were randomly obtained from GBM patients during tumor removal. Written informed consent was obtained from all patients for sample collection and research purposes. The study was conducted in accordance with the ethical standards and guidelines of the Institutional Review Board (IRB No. 4–2021–1319) and complied with all applicable institutional and national regulations.

Three GBM TSs (TS13–64, TS13–30, and TS15–88) were individually isolated from tissue specimens obtained from three separate GBM patients and cultured in TS complete medium comprising Dulbecco’s Modified Eagle’s Medium/ nutrient mixture F-12 (DMEM/F-12; Mediatech, Manassas, VA, USA), supplemented with 20 ng/mL epidermal growth factor (EGF; Novoprotein, Summit, NJ, USA), 20 ng/mL basic fibroblast growth factor (bFGF; Novoprotein), 50 U/mL penicillin, and 50 μg/mL streptomycin and 1 × B27 (Invitrogen, San Diego, *CA*, USA) [14,18,30].

Normal human astrocytes (NHA) were obtained commercially (Lonza, Walkersville, MD, USA) and cultured in Astrocyte Basal Medium supplemented with the Astrocyte Growth Kit (Lonza). Cells were. All *in vitro* experiments were maintained at 37 °C in a humidified incubator with 5 % CO₂, and the medium was replaced every 3–4 days.

Luc-TS13–64 was generated by incorporating CMV-firefly luciferase lentivirus (Cellomics Technology, Thermo Fisher Scientific, Pittsburg, PA, USA) into GBM TSs followed by puromycin selection.

### Synthesis of BTK inhibitors

The used BTK inhibitors (SPA1758, SPA1763, SPA8004, SPA8007, SPA8009) have been previously reported in two scientific publications [2,24]. In brief, for pyranochromenone analogs (SPA1758, SPA1763), electrophilic warheads were introduced to decursinol via a dicyclohexylcarbodiimide coupling reaction. For tetrahydroisoquinoline-linked aminopyridine analogs (SPA8004, SPA8007, SPA8009), tetrahydroisoquinoline intermediate was prepared via Bischler–Napieralski cyclization and reduction with lithium aluminum hydride. The purposed compounds were obtained through *N*-alkylation, nucleophilic substitution reactions, and nitro group reduction under mild acidic conditions.

### Cell viability and ATP level assays

GBM TSs were dissociated into single cells and plated into a transparent 96-well plate for cell viability assays and a black 96-well plate for ATP assays at a density of 1 × 10^4^ cells per well. After 24 h of incubation, BTK inhibitors (SPA1758, SPA1763, SPA8004, SPA8007, SPA8009) were added and incubated for an additional 72 h at 37 ℃. The proliferative effect of BTK inhibitors on GBM TSs was assessed using Cell Counting Kit-8 (Dongin, Seoul, Korea). Ten microliters of WST reagent were added to each well, and absorbance at 450 nm was measured after 1 h of incubation at 37 ℃. ATP levels were measured using the Cell Titer-Glo Luminescent Cell Viability Assay kit (Promega, Fitchburg, WI, USA) following the manufacturer’s protocol. One hundred microliters of Cell Titer-Glo was added to each well, and luminescence was measured using a Centro XS^3^ LB 960 Spectrometer (PerkinElmer, Waltham, MA, USA) after 10 min of incubation at room temperature. Each experiment was conducted in triplicate, and the cell viability results were expressed as the percentage of viable cells relative to that in the control.

### Determination of inhibitory concentrations

Cell viability was assessed using WST after treatment with increasing concentrations of each compound (0–100 μM) for 72 h. The viability at each concentration was normalized to that in the untreated control (set as 100 %). Dose–response curves were generated and fitted using non-linear regression with a four-parameter logistic model (variable slope) in the GraphPad Prism 9 software (GraphPad Software, San Diego, *CA*, USA). The concentration necessary to inhibit cell viability by 20 % (IC_20_) and 50 % (IC_50_) was calculated based on the fitted curve by determining the drug concentration corresponding to 80 and 50 % viability, respectively.

### Neurosphere formation assay

GBM TSs were dissociated, and ten single cells were seeded into each well of a transparent 96-well plate. Following 24 h of incubation at 37 ℃, 10 μM BTK inhibitors (SPA8007, SPA8009) were introduced to the wells. After 3 weeks of incubation, the number of wells with sphere formation was counted and the results were expressed relative to the number of wells with sphere formation in the control group. Additionally, the average radius of the spheres in each experimental group was measured using the ToupView software (x64 v.3.7.1460, ToupTek Photonics, Zhejiang, China).

### Three‑dimensional (3D) invasion assays

A Matrigel mixture comprising TS complete medium, Matrigel (Corning Life Sciences, Tewksbury, MA, USA), and rat tail collagen type I (BD Biosciences, Franklin Lakes, NJ, USA) was dispensed into each well of a transparent 96-well plate. Prior to gelation, single GBM TS spheroids were seeded into each matrix-filled well and incubated at 37 ℃ for 30 min. Subsequently, TS complete medium containing BTK inhibitors (SPA8007, SPA8009) was added to each well and incubated for 72 h. The invaded area was measured using the ToupView image analysis software (ToupTek Photonics) and calculated by relating this area to that at 0 h.

### Western blot

Dissociated 4 × 10⁵ single cells were seeded into a 100-mm culture dish and incubated for 24 h. Following this incubation period, 10 μM BTK inhibitors (SPA8007, SPA8009) were introduced. After additional 72 h of incubation, TSs were harvested and collected using sedimentation, followed by protein extraction utilizing cell extraction buffer (Invitrogen) supplemented with 1× protease and phosphatase inhibitor cocktail (Thermo Fisher Scientific, Waltham, MA, USA). GBM TS lysates were then subjected to SDS-polyacrylamide gel electrophoresis. The separated proteins were transferred onto nitrocellulose membranes (GE Healthcare Life Sciences, Little Chalfont, UK) and incubated in 3 % bovine serum albumin blocking solution for 1 h at room temperature and incubated overnight at 4 °C with specific primary antibodies with 1:1000 dilution of SOX2, β-catenin (Cell Signaling Technology, Danvers, MA, USA), PDPN, GAPDH (Santa Cruz Biotechnology, Santa Cruz, *CA*, USA), Nestin, N-cadherin (Sigma-Aldrich, St. Louis, MO, USA) and ZEB1 (Abcam, Cambridge, UK). Secondary horseradish peroxidase-conjugated IgG antibodies (Santa Cruz Biotechnology) were introduced and allowed to incubate for 1 h at room temperature. Western Lightning Plus-enhanced chemiluminescence reagent (PerkinElmer) was used for detection, and an ImageQuant LAS 4000 mini (GE Healthcare Life Sciences) was employed to capture the images. Western blot images were minimally processed to improve clarity while maintaining data integrity. In certain experiments, lanes corresponding to unrelated treatment conditions (*e.g.*, Ibrutinib) were excluded to enhance focus and clarity; such modifications are explicitly noted in the respective figure legends. Where applicable (Figs. 2b and 3b), splicing boundaries are clearly marked by thin vertical lines. The corresponding uncropped and unprocessed blots are provided in **Supplementary Figs. 5** and **6**.

### Bulk RNA sequencing

For TS bulk RNA sequencing, single 4 × 10⁵ cells were seeded in 100-mm culture dishes. After 24 h of incubation, 10 μM BTK inhibitors (SPA8007, SPA8009) were added and incubated for an additional 72 h. TSs were then harvested and treated with Trizol for RNA extraction. Total RNA samples were assessed using an Agilent 2100 Bioanalyzer (Agilent Technologies, Santa Clara, *CA*, USA). Fastq files were generated using bcl2fastq (v2.2) and quality was checked using FastQC (v.0.11.9). Next, the Illumina TruSeq adapter sequences were trimmed out from the read files using Skewer (v.0.2.2) with paired-end mode default parameters. The reads were mapped to the human reference genome (GRCh38) using STAR aligner (v.2.7.10a). Gene counts were normalized to transcripts per million criteria with R. The cell effect was corrected using the sva R package and genes with a log2-fold change > 0.5 and an adjusted *p*-value < 0.05 were selected as differentially expressed genes (DEGs). DEG analyses were conducted using DESeq2 R package [27] and visualized using the pheatmap R package. To find associations of genes and biological pathway terms, DEG lists were run using the enrichR R package. Gene Ontology Biological Process 2023 was used as the reference term set (accession number: GSE288022).

### Mouse orthotopic xenograft model

*In vivo* experiments and animal care were approved by the Committee for the Care and Use of Laboratory Animals at Yonsei University College of Medicine (approval no. 2023–0237) and were conducted following the guidelines established by the US National Institutes of Health. The reporting of animal experiments in this study adheres to the ARRIVE guidelines.

Male athymic nude mice aged 6–8 weeks (Central Lab. Animal Inc., Seoul, Korea) were used after 1 week of acclimatization under controlled environmental conditions, including humidity (55 ± 5), lighting (12-h light/ dark cycle), and temperature (22 ± 2 °C). Luc-TS13–64 cells were dissociated and plated in a 100-mm culture plate and treated with 10 μM of SPA8007 for 72 h following a standardized protocol [5,19,26,28,29,36,37,45,49]. Subsequently, 1 × 10⁵ cells/mouse were injected into the right frontal lobe of mice using a guide-screw system [21], ensuring an injection depth of 4.5 mm. Mice with 15 % body weight reduction were euthanized following the guidelines of the American Veterinary Medical Association, which was reflected in the survival curve.

### Bioluminescence imaging

The mice were anesthetized with 2.5 % isoflurane, and 100 μl of d-Luciferin / 20 g of mouse (30 mg/ml; dissolved in DPBS, Promega) was administered via the intraperitoneal route 10 to 15 min prior to bioluminescence image acquisition. Images were captured using an IVIS imaging system and the Living Image v4.2 software (Caliper Life Sciences, Hopkinton, MA, USA).

### Immunohistochemistry

Paraffin-embedded brain tissue blocks were cut at a thickness of 4 μm using a microtome and mounted onto adhesive slides. An automated instrument (Discovery XT, Ventana Medical Systems, Tucson, AZ, USA) was employed for antigen retrieval and antibody binding, while a peroxidase/3,3ʹ-diaminobenzidine staining system was utilized to detect Nestin and ZEB1.

### Statistical analysis

Significant differences between the control and treatment groups were calculated using one-way analysis of variance with Tukey’s post hoc test for multiple comparisons. Survival analysis was conducted utilizing the Kaplan-Meier method and comparisons performed through log-rank tests. All graphical and statistical analyses were performed using GraphPad Prism 9 software (GraphPad Software Inc., San Diego, *CA*, USA), with *p*-values < 0.05 (*), < 0.01 (**), and < 0.001 (***) considered statistically significant.

## Results

### BTK expression levels are significantly elevated in both GBM tissues and TSs compared to normal brain tissues and normal human astrocytes

**Fig. 1:**
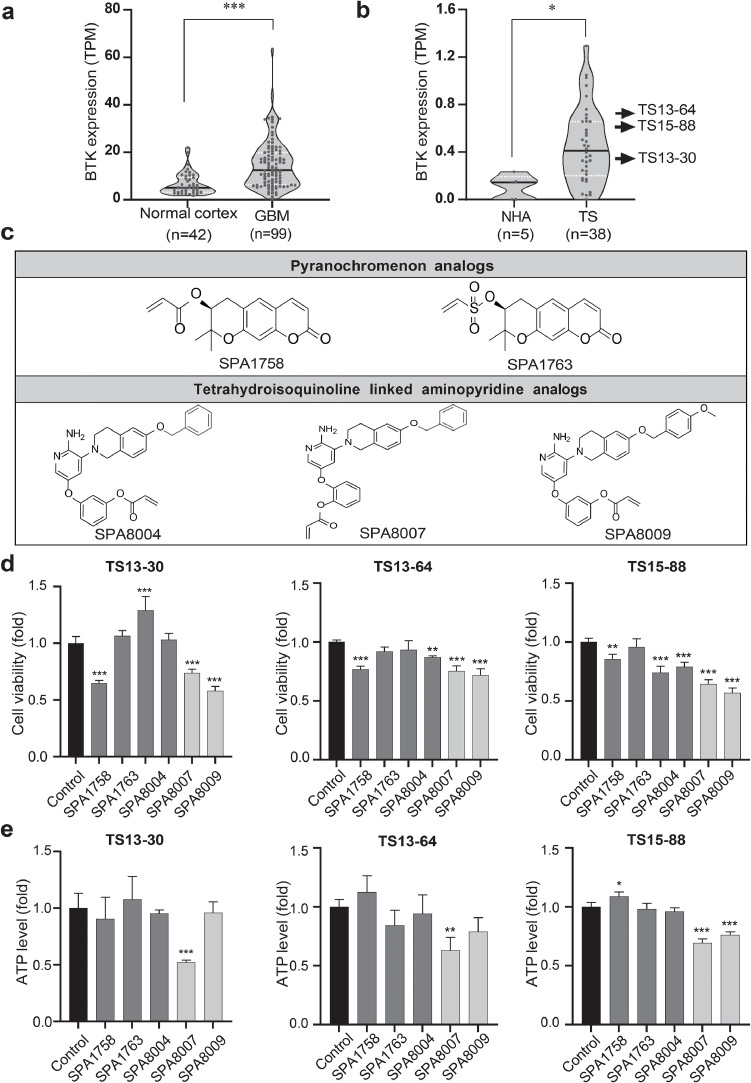
**BTK expression in both GBM tissues and TSs and anti-proliferative effects of new BTK inhibitors**. **a** mRNA expression level of BTK in tumor free cortex tissue (*n* = 42) and GBM tumor tissue (*n* = 99) obtained from tissue bulk RNA sequencing of severance patients. **b** mRNA expression level of BTK in normal human astrocyte (NHA, *n* = 5) and TSs (*n* = 38) derived from patient tumor tissue. **c** Chemical structure of five newly synthesized BTK inhibitors. **d** Cell viability was measured after treatment of three TSs with the five new BTK inhibitors. **e** Cell ATP levels were measured after treatment of three TSs with the five new BTK inhibitors. * *p* < 0.05, ** *p* < 0.01, and *** *p* < 0.001 compared with the control.

Bulk RNA sequencing analysis of tissues from primary GBM patients revealed markedly increased (*p* < 0.001) BTK expression levels in tumor tissues (*n* = 99) compared to tumor-free cortex tissues (*n* = 42**;** Fig. 1a) consistent with prior studies [17,43]. **Supplementary Table 1** and **supplementary Table 2** contain detailed clinical and histopathological information regarding tumor-free cortex and tumor tissues, respectively**.** Additionally, significantly elevated (*p* < 0.05) BTK expression was observed in GBM TSs isolated from the bulk tumor compared to its tumor-free counterpart, NHA (Fig. 1b) [3,4,52]. Three representative GBM TSs (TS13–30, TS13–64, TS15–88) were selected for further investigation. **Supplementary Table 3** shows detailed histopathological characteristics of GBM tissues and corresponding TSs. Brief information of the five newly developed BTK inhibitors (SPA1758, SPA1763, SPA8004, SPA8007, and SPA8009), validated for their efficacy in earlier investigations (Fig. 1c), is provided in **supplementary Table 4**.

### SPA8007 and SPA8009 exhibit superior cytotoxic effects in TSs relative to other compounds

To identify the most promising candidate drug, WST and ATP assays were performed. Three GBM TSs were treated with each inhibitor for 72 h at increasing doses to determine the appropriate concentration that induces cellular phenomena with minimal effects on cell viability (IC_20_). Sub-cytotoxic concentrations are widely recommended for assessing stemness and invasion phenotypes to avoid confounding apoptotic responses [4,8,13,37]. Based on this rationale, 10 μM was selected as the experimental concentration for all subsequent assays (**Supplementary Figs. 1, 2 and Supplementary Table 5)**. Among the tested compounds, SPA8007 and SPA8009 demonstrated the highest cytotoxic effects, whereas SPA1758, SPA1763, and SPA8004 exhibited minimal cytotoxicity across all three TSs (Fig. 1d). However, in the ATP assay, only SPA8007 reduced ATP levels significantly (*p* < 0.01) in all three TSs (Fig. 1e). Consequently, SPA8007 and SPA8009 were chosen for further investigation.

To evaluate tumor selectivity and potential safety, IC_20 and_ IC_50_ values of SPA8007, SPA8009, and ibrutinib in NHA and the three GBM TSs were determined and compared **(Supplementary Fig. 2 and Supplementary Table 5)**. SPA8007 exhibited comparable IC_20_ values in NHA (9.7 μM) and TS13–64 (10 μM), and SPA8009 showed markedly reduced toxicity in NHA (44 μM) while retaining strong activity in TS13–64 (0.4 μM) whereas ibrutinib was more toxic to NHA (9.2 μM) than to TS13–64 (19 μM) with *p value of < 0.0001*.

Collectively, these findings indicated that the novel BTK inhibitors SPA8007 and SPA8009 induce greater cell death in GBM TSs than the other three inhibitors and offers a favorable balance of potency and selectivity for therapeutic application than ibrutinib.

### SPA8007 and SPA8009 significantly reduce the stemness and invasive properties of TSs

**Fig. 2:**
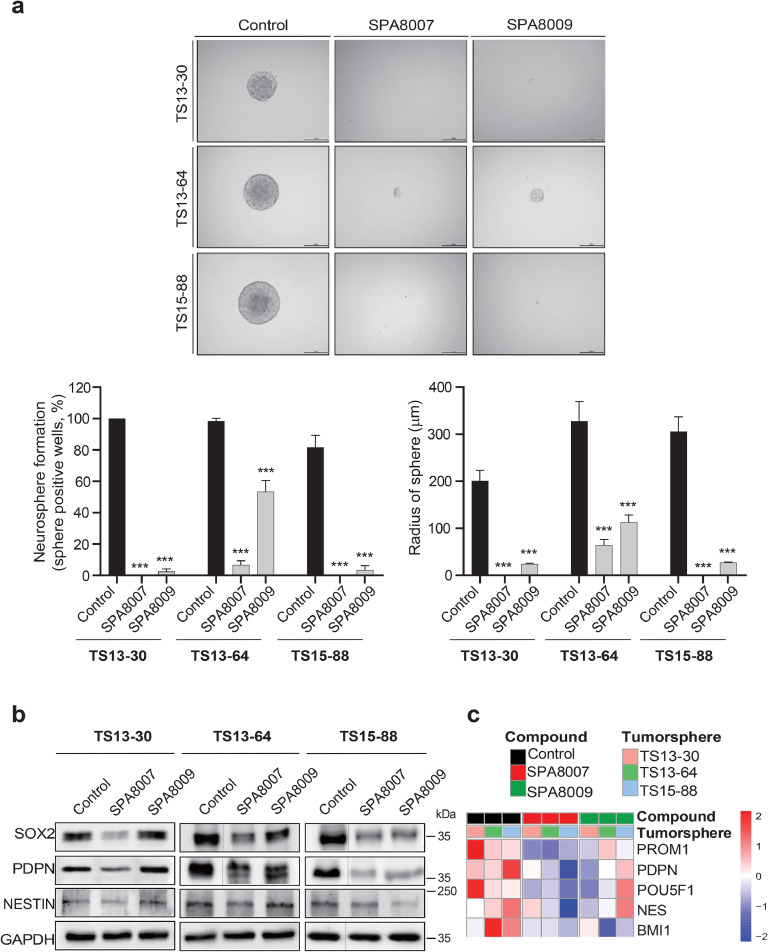
**Effect of SPA8007 and SPA8009 on stemness of GBM TSs**. **a** The abundance of sphere-positive wells were determined after treatment with SPA8007 (10 μM) and SPA8009 (10 μM) relative to the control in three TSs. Changes in sphere radii were evaluated after treatment with SPA8007 (10 μM) and SPA8009 (10 μM) relative to the control in three TSs. **b** Western blot and **c** heatmaps showing changes in stemness-related markers after treatment with SPA8007 and SPA8009 relative to the control in three TSs. Initially, lanes corresponding to Control, Ibrutinib, SPA8007, and SPA8009 were loaded; the ibrutinib lane was excluded for clarity, as indicated by a thin vertical line separating Control and SPA8007. The uncropped, original immunoblots are provided in **Supplementary Fig. 5**. Differences among groups were compared using one-way ANOVA with Tukey’s post hoc test. * *p* < 0.05, ** *p* < 0.01, and *** *p* < 0.001 compared with the control.

**Fig. 3:**
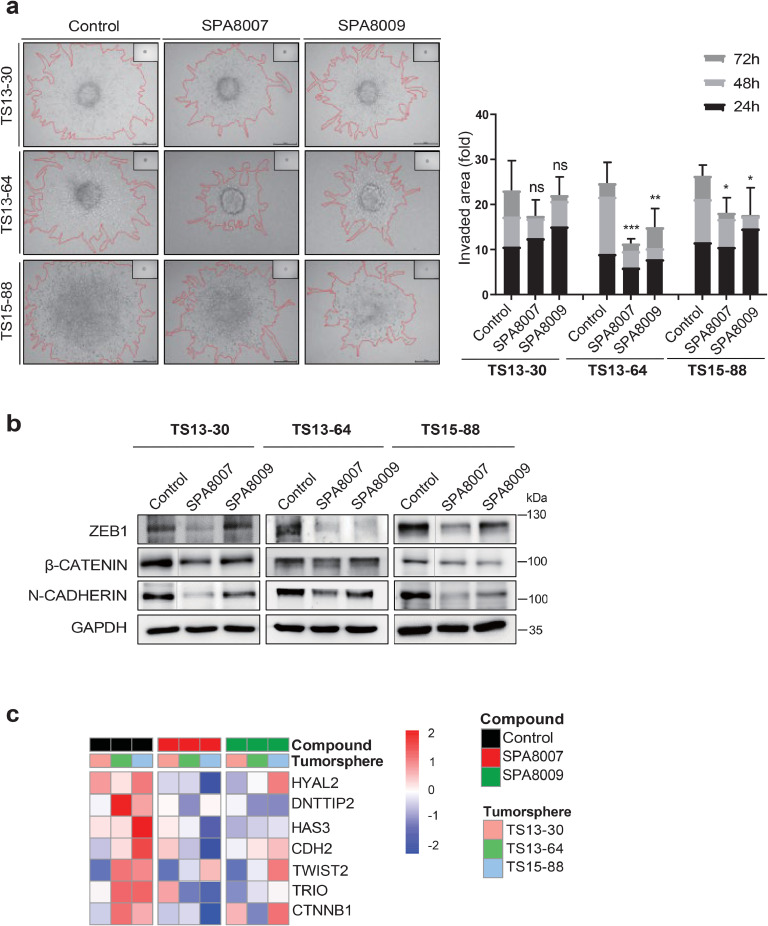
**Effect of SPA8007 and SPA8009 on invasiveness of GBM TSs**. **a** Invasiveness of three TSs was evaluated in a Matrigel/collagen matrix after treatment with SPA8007 (10 μM) and SPA8009 (10 μM) for 72 h. **b** Western blot and **c** RNA sequencing data displaying invasiveness-related marker changes after SPA8007 (10 μM) and, SPA8009 (10 μM) treatment for 72 h. Initially, lanes corresponding to Control, Ibrutinib, SPA8007, and SPA8009 were loaded; the ibrutinib lane was excluded for clarity, as indicated by a thin vertical line separating Control and SPA8007. The uncropped, original immunoblots are provided in **Supplementary Fig. 6**. Differences among groups were compared using one-way ANOVA with Tukey’s post hoc test. * *p* < 0.05, ** *p* < 0.01, and *** *p* < 0.001 compared with the control.

The reduction in the stemness and invasive potential of the selected drugs in TSs was evaluated using neurosphere formation and 3D invasion assays, respectively. Both SPA8007 and SPA8009 significantly decreased (*p* < 0.001) the percentage of wells exhibiting positive spheres and reduced (*p* < 0.001) the sphere radius across all three TSs compared to the respective control groups (Fig. 2a). Additionally, SPA8007 substantially reduced the stemness-related marker proteins SOX2 and PDPN in all three TSs, while SPA8009 reduced SOX2 and PDPN in TS13–64 and TS15–88, as evidenced by western blot analysis (Fig. 2b). Moreover, bulk RNA sequencing data showed that both SPA8007 and SPA8009 reduced the number of stemness-related genes (Fig. 2c). Combined, these data support that these two compounds suppress the stemness of GBM TSs. In the 3D invasion assay, both SPA8007 and SPA8009 significantly decreased (*p* < 0.05) the invasion capacity of TS13–64 and TS15–88 compared to their respective control groups (Fig. 3a). Western blot analysis demonstrated that SPA8007 significantly reduced the expression of invasiveness-related marker proteins, ZEB1, β-catenin, and N-cadherin, in TS13–30, while in TS13–64 and TS15–88, only ZEB1 and N-cadherin markers were reduced. While SPA8009 substantially suppressed ZEB1, β-catenin, and N-cadherin markers in TS15–88, it only suppressed the ZEB1 marker in TS13–64 (Fig. 3b). Additionally, bulk RNA sequencing data showed that both SPA8007 and SPA8009 reduced other invasiveness-related markers (Fig. 3c). These results demonstrate that SPA8007 and SPA8009 effectively inhibit the invasive properties of GBM TSs.

### SPA8007 significantly increases the survival rate in a mouse xenograft model

**Fig. 4:**
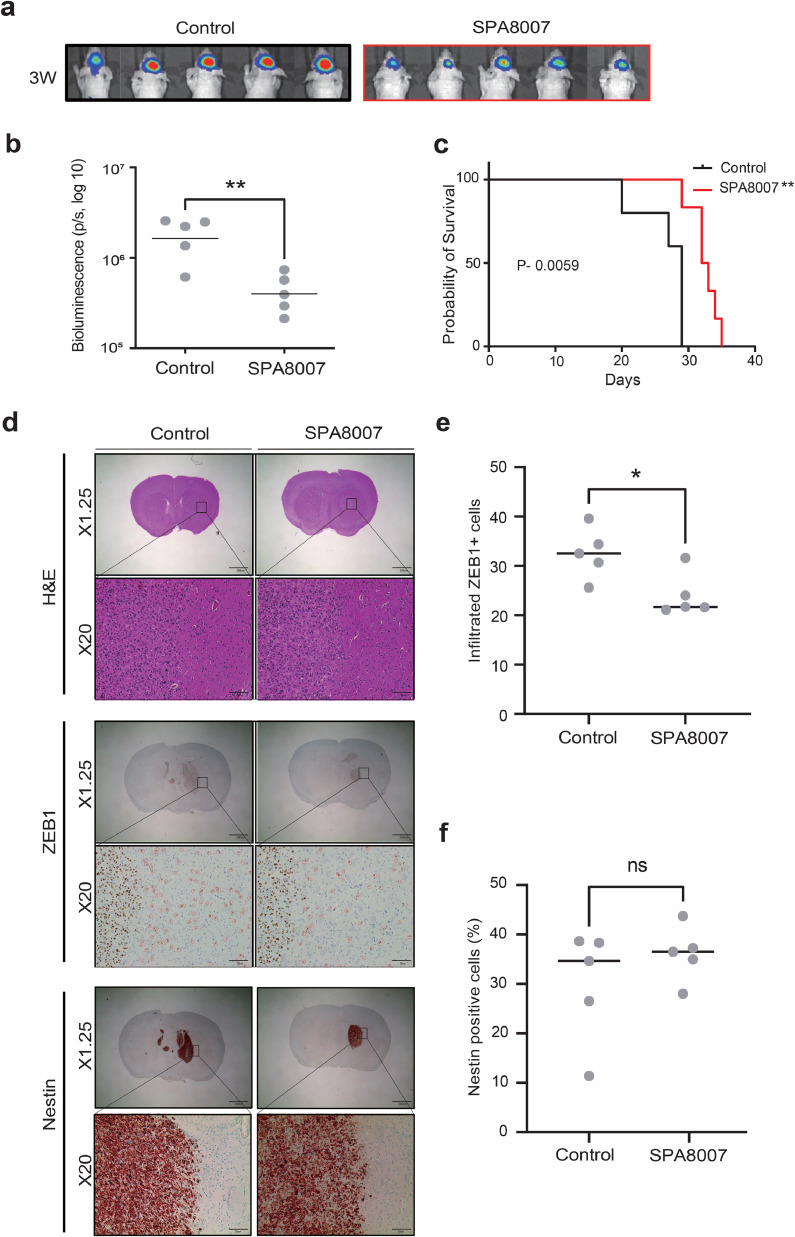
**Therapeutic effect of SPA8007 in a mouse orthotopic xenograft model**. Luc TS13–64 TSs were treated with SPA8007 (10 μM) for 72 h and harvested viable TSs were injected into the mouse cortex (Control: *n* = 5, SPA8007: *n* = 5). **a, b** Tumor volume was assessed through bioluminescence imaging as total photon flux. **c** Kaplan Meier analysis showing the survival probability of the treated group *vs.* the control group of mice. **d** H&E staining showing representative brains for each group. IHC analysis was performed in mouse brain sections for evaluation of Nestin and ZEB1 expression levels. **e** Comparison of infiltrated ZEB1^+^ cells in control and SPA8007-treated groups. **f** Comparison of Nestin positive cells in control and SPA8007-treated groups.

In the mouse orthotopic model, tumor growth in TS13–64, treated with SPA8007, was compared to that in the TS13–64 control group. Bioluminescence intensity, estimated based on total flux (photon/s) 3 weeks after injection of cells, indicated that the SPA8007 group exhibited significantly smaller (*p* < 0.01) tumor sizes than the control group (Fig. 4a and b), suggesting that treatment with SPA8007 reduced the ability of GBM TSs to promote tumor growth in the mouse model. Furthermore, Kaplan-Meier survival analysis demonstrated that TS13–64 treated with SPA8007 exhibited significantly prolonged (*p* < 0.0059) survival compared to the control group (Fig. 4c). Immunohistochemistry analysis revealed significantly reduced (*p* < 0.05) expression levels of the invasiveness marker ZEB1 in the SPA8007-treated group compared to the control (Fig. 4d and e). However, Nestin expression was not significantly altered in the SPA8007-treated group (Fig. 4f). Collectively, these results demonstrate that SPA8007 reduced the tumorigenic capacity of TS13–64.

## Discussion

GBM remains an incurable malignancy, highlighting the urgent need for identifying novel therapeutic targets [7,31]. Previous studies have identified elevated BTK expression in GBM tumors [7,17,31,43], consistent with our findings based on the Severance cohort (Fig. 1a), suggesting that BTK could be a promising therapeutic target. However, the use of BTK inhibitors to treat GBM has been hampered by their significant toxicity [6,40], leading to the termination of many clinical studies [54]. The toxicity of ibrutinib stems from its covalent binding to a conserved cysteine residue in the ATP-binding domain of non-BTK tyrosine kinases [1]. It thus inhibits multiple off-target kinases, including ERBB family members (EGFR, HER2, HER3, HER4), TEC family kinases (TEC, BTK, BMX, ITK, RLK), and JAK3. Notably, at 1 μM/L, ibrutinib inhibits over 95 % of 16 kinases [41], highlighting its broad activity and the challenge of reducing off-target effects while preserving efficacy. To address this issue, we developed more selective and less toxic BTK inhibitors, *i.e.*, SPA1758, SPA1763, SPA8004, SPA8007, and SPA8009 [2,24]. The pyranochromenone analogs SPA1758 and SPA1763 exhibited exceptional BTK inhibitory activity (98.2 % and 95.8 %, respectively) while maintaining minimal off-target inhibition against ITK (17.3 %), EGFRK (32.9 %), and JAK3 (13.2 %), and showing no activity against ERBB4 [2]. Additionally, the aminopyridine derivatives SPA8004, SPA8007, and SPA8009 demonstrated similarly potent BTK inhibition (97 %, 98 %, and 92 %, respectively) with low inhibition against EGFRK, ITK, TEC, and TXK [24]. Moreover, these compounds were validated *in vivo* in a murine inflammatory model, where they significantly reduced (*p* < 0.001) collagen-induced arthritis severity in a dose-dependent manner without observable toxicity. In a hematological malignancy model, they achieved a 47 % reduction in tumor size compared to that in controls, without adverse clinical symptoms, significant weight loss, or evidence of liver or kidney toxicity upon necropsy [2,24]. These previous observations highlight the therapeutic potential of the tested inhibitors as safer, more selective alternatives for targeting BTK-driven diseases.

Among the 38 GBM-TSs isolated, TS13–30, TS13–64, and TS15–88 were chosen for their superior proliferative capacity [36]. Elevated BTK expression in these TSs, which matches the high BTK expression of their corresponding tumor tissues (Fig. 1a and b), confirmed that they accurately reflect the molecular and functional characteristics of GBM and are a suitable model for evaluating drug efficacy. Our findings revealed that the newly synthesized SPA8007 and SPA8009 significantly reduced (*p* < 0.001) the proliferative capacity of GBM TSs (Fig. 1d and e) and downregulated pathways crucial for the cellular biosynthesis (**Supplementary Fig. 3**). This finding coincides with the anti-proliferative effect of ibrutinib in GBM and ovarian cancer cell lines [43,53,55]. Another study showed that both ibrutinib treatment and BTK gene silencing inhibited the self-renewal ability of CSCs in ovarian cancer cell lines [55]. Similarly, we also observed nearly complete inhibition (*p* < 0.001) of sphere formation capacity after treatment with SPA8007and SPA8009 in all three TSs after 3 weeks of drug treatment (Fig. 2a). Western blot and transcriptomic analysis further supported these results, showing substantial reductions in the expression of stemness markers, including SOX2, PDPN, and Nestin (Fig. 2b and c). Given the critical role of TSs in driving therapy resistance and recurrence, our results suggest that targeting stemness with the two inhibitors could represent a promising therapeutic strategy for GBM patients [16,18]. In addition to their effects on stemness, SPA8007 and SPA8009 also significantly inhibited (*p* < 0.05) GBM TS invasion, which is consistent with the anti-invasive effect of ibrutinib in GBM cell lines reported previously [43,53]. Evaluating invasiveness in TSs is a critical factor in GBM research, as numerous studies have highlighted that tumor invasion is predominantly driven by the CSC subpopulation [20,48]. Using a physiologically relevant 3D invasion assay, we observed that SPA8007 and SPA8009 markedly reduced (*p* < 0.05) radial invasion of GBM TSs within 3 days of treatment, without broad cytotoxicity (Fig. 3a). Western blot analysis further validated these findings, revealing significant downregulation of genes associated with invasiveness such as ZEB1, β-catenin, and N-cadherin (Fig. 3b). This molecular evidence coincides with our transcriptomic analysis (Fig. 3c) and highlights the potential of SPA8007 and SPA8009 to disrupt processes critical to GBM progression. Moreover, in an orthotopic xenograft model, treatment with SPA8007 significantly decreased tumor sizes (*p* < 0.01; Fig. 4a and b) and prolonged survival (*p* = 0.0059), as demonstrated by Kaplan–Meier analysis (Fig. 4c), further highlighting its therapeutic potential. SPA8007 was selected for *in vivo* study because it showed greater anti-stemness and anti-invasiveness activity than SPA8009. TS13–64 cells were singled out from the three TSs in the *in vivo* study because of their high BTK expression levels (Fig. 1b), their notably brief mouse survival time, and their pronounced aggressiveness within the mouse brain environment [37]. To evaluate the intrinsic effects of SPA8007 on tumor cells independently of systemic pharmacokinetic factors, SPA8007 was administered as a pre-treatment to the cells prior to implantation. This approach was chosen because ibrutinib, the parent compound of SPA8007 and SPA8009, has been reported to exhibit very limited penetration into the central nervous system despite feasible blood brain barrier permeability due to active efflux mechanisms that substantially restrict its brain exposure [42]. Furthermore, since SPA8007 and SPA8009 are novel compounds with limited pharmacokinetic data available, the pre-treatment method was deemed appropriate for this *in vivo* study. Immunohistochemistry analysis was conducted on mouse brain tissue to evaluate the impact of SPA8007 on the local microenvironment, revealing a significant reduction (*p* < 0.05) in the levels of invasiveness markers in brains treated with SPA8007 compared to those in control mouse brains (Fig. 4c and d). Ibrutinib, however, failed to show any therapeutic effect in the *in vivo* setting (**Supplementary Fig. 4).** As these results did not demonstrate statistically significant efficacy, we opted to present this data in the **Supplementary** section.

Combined, these findings highlight the anticancer potential of SPA8007 and SPA8009 in human GBM cells. Given that targeting stemness and invasiveness has been recognized as a critical therapeutic approach for GBM, the outstanding performance of SPA8007 and SPA8009 in these aspects, along with their impact on survival, positions them as promising strategies to curb the uncontrolled proliferation of GBM.

This study has several limitations, including the use of a single *in vivo* model. Future studies should employ multiple TS model *in vivo* to better assess the efficacy and safety profile of the tested inhibitors and to validate generalizability. Moreover, the lack of systemic drug administration is another limitation of this study. Future work should include pharmacokinetic analyses and develop strategies to enhance brain distribution and absorption to obtain clinically relevant data. Finally, considering the critical role of the origin of GBM cells, our findings underscore the need to evaluate the cytotoxic effect of SPA8007 on these cells to further elucidate its therapeutic potential in targeting GBM at its source [25,51].

## Funding

This work was supported by the National Research Foundation of Korea (NRF) grants funded by the Korean government (MSIT; RS-2025–00,523,374), the 10.13039/501100003725National Research Foundation of Korea (NRF) grant funded by the Korea government (MSIT; RS-2022-NR067592), Korea Health Technology R&D Project through the Korea Health Industry Development Institute (KHIDI) by the Ministry of Health & Welfare, Republic of Korea (RS-2024–00,438,443), Bio&Medical Technology Development Program of the NRF by the Korean government (MSIT; RS-2024–00,437,820), NRF of Korea grant funded by the Korea government (MSIT; RS-2024–00,408,191), and an NRF grant funded by the Korean government (MSIT; No.2021R1A2C1003358).

## Data availability

The sequencing data described in this paper are available on Gene Expression Omnibus (accession number: GSE288022).

## CRediT authorship contribution statement

**Euna Jo:** Writing – review & editing, Writing – original draft, Visualization, Validation, Software, Methodology, Investigation, Formal analysis, Data curation. **Eun Lee:** Visualization, Validation, Software, Methodology, Investigation, Formal analysis, Data curation, Conceptualization. **Yoojung Oh:** Visualization, Validation, Software, Methodology, Investigation, Formal analysis, Data curation. **Dongkyu Lee:** Visualization, Validation, Software, Methodology, Investigation, Formal analysis, Data curation, Conceptualization. **Byungho Lee:** Visualization, Validation, Software, Methodology. **Kibyeong Kim:** Visualization, Validation, Software, Methodology. **Ran Joo Choi:** Visualization, Validation, Software, Methodology. **Jiyun Hong:** Visualization, Validation, Software, Methodology. **Yuesong Jeon:** Visualization, Validation, Software, Methodology. **Hyewon Cho:** Visualization, Validation, Software, Methodology. **Yong-Sung Choi:** Visualization, Validation, Software, Methodology. **Sangwoo Kim:** Supervision, Resources. **So Young Won:** Visualization, Validation, Software, Methodology. **Seonah Choi:** Supervision, Resources. **Tae Hoon Roh:** Supervision, Resources. **Ju Hyung Moon:** Supervision, Resources. **Eui Hyun Kim:** Supervision, Resources. **Jong Hee Chang:** Supervision, Resources. **Raok Jeon:** Supervision, Resources, Investigation, Formal analysis, Data curation, Conceptualization. **Seok-Gu Kang:** Writing – review & editing, Supervision, Funding acquisition, Conceptualization.

## Declaration of competing interest

The authors have no conflicts of interest to declare.

## Appendix Supplementary materials

Image, application 1
