# Supplementary material for: The new Bruton’s tyrosine kinase inhibitors SPA8007 and SPA8009 reduce stemness and invasiveness of patient-derived glioblastoma tumorspheres
Source: Transl Oncol. 2025 Oct 26;63:102585. doi: 10.1016/j.tranon.2025.102585 (PMC12595426; doi:10.1016/j.tranon.2025.102585)
Supplement: Supplementary file 1 [file mmc1.docx]

**Supplementary Fig. 1**

**
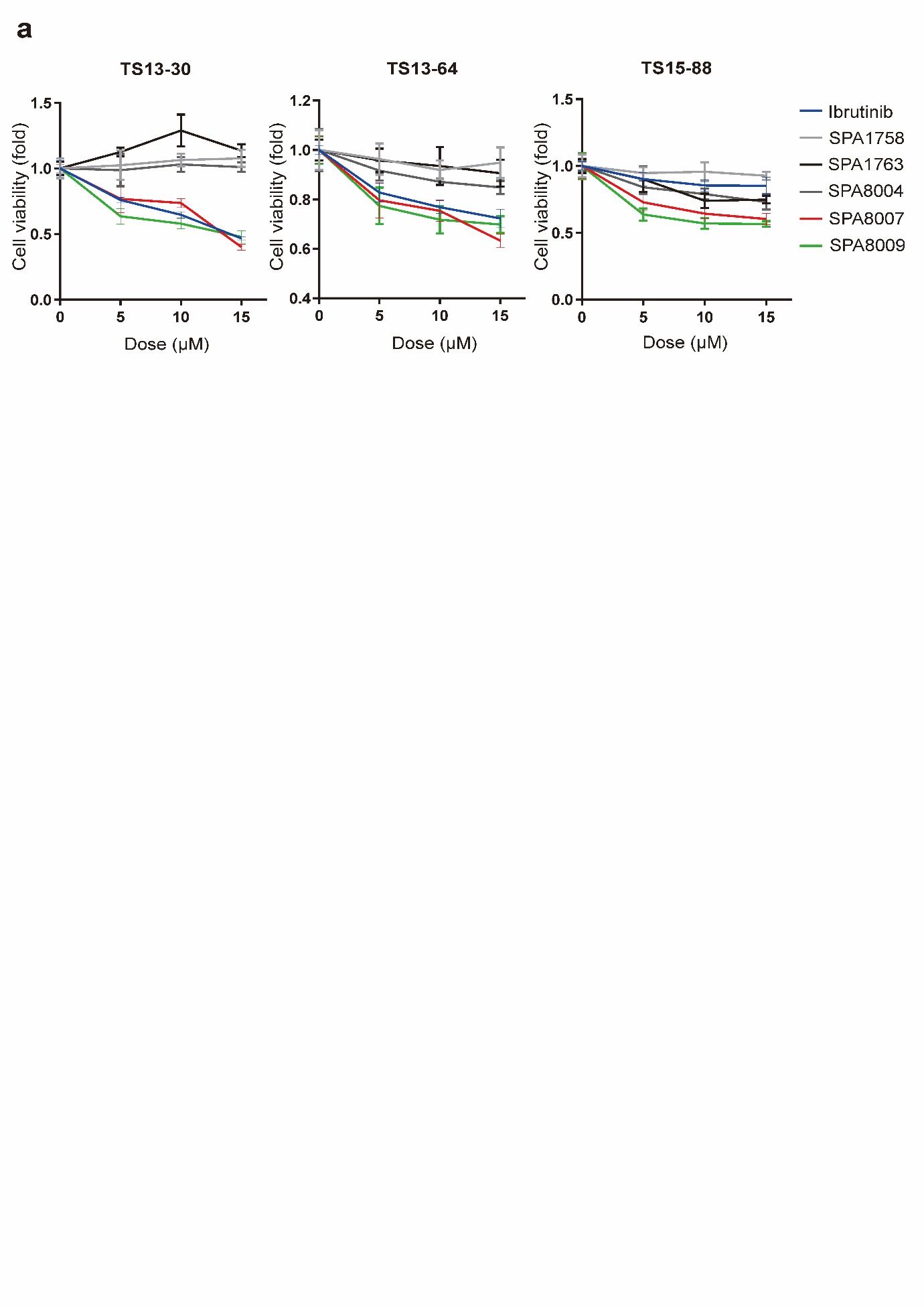
**

**Supplementary Fig. 2**

**
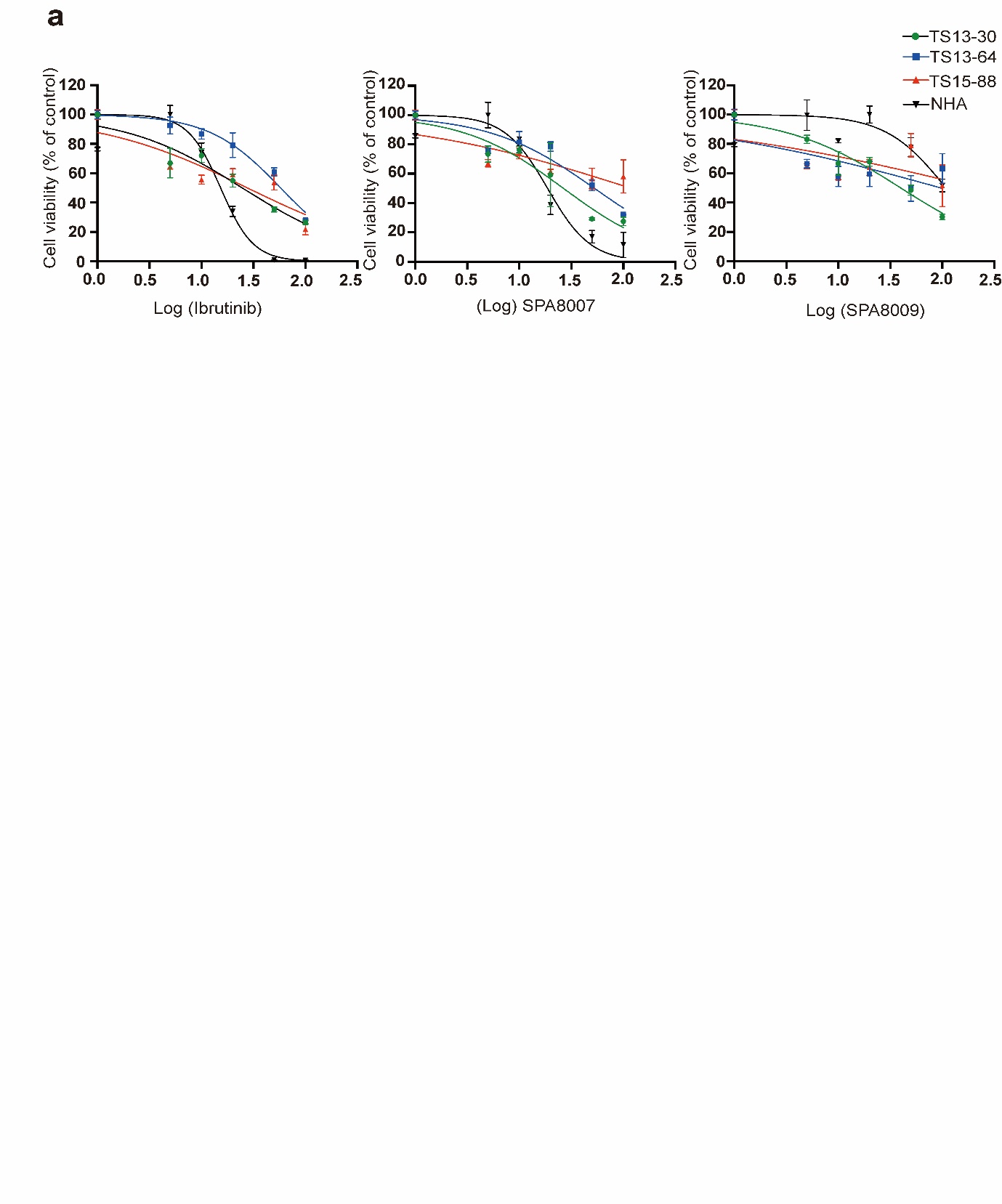
**

**Supplementary Fig. 3**

**
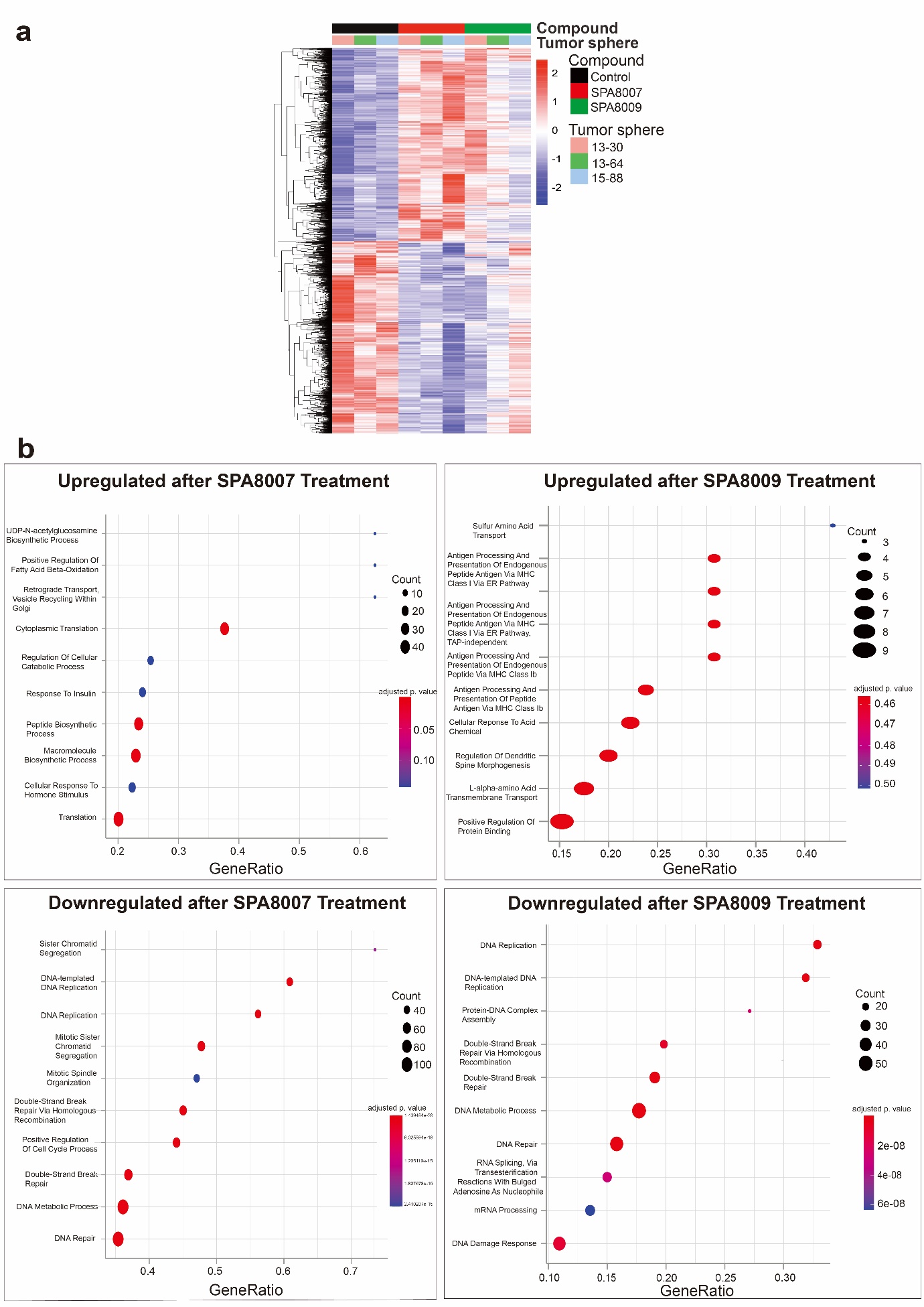
**

**Supplementary Fig. 4**

**
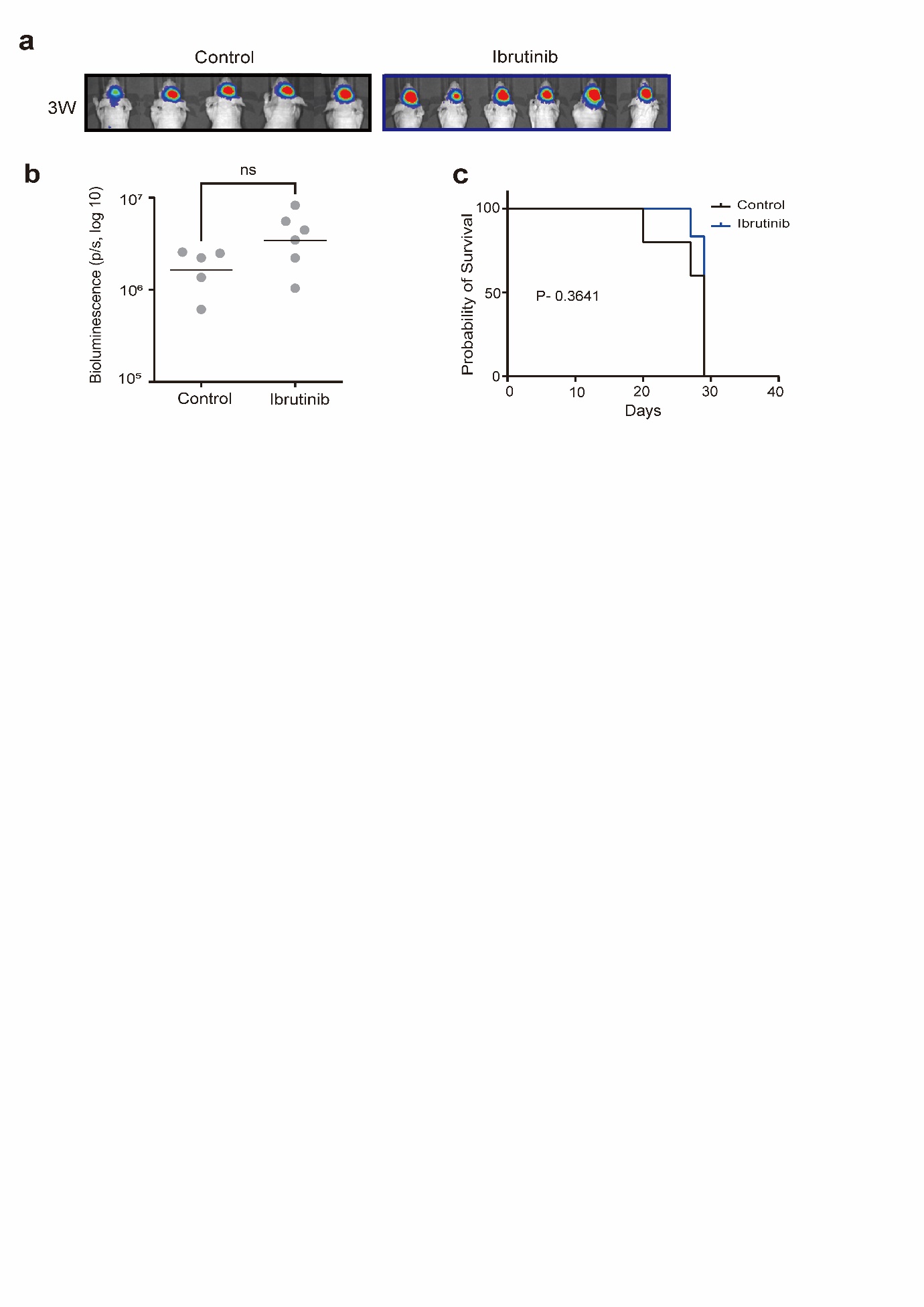
**

**Supplementary Fig. 5**

**
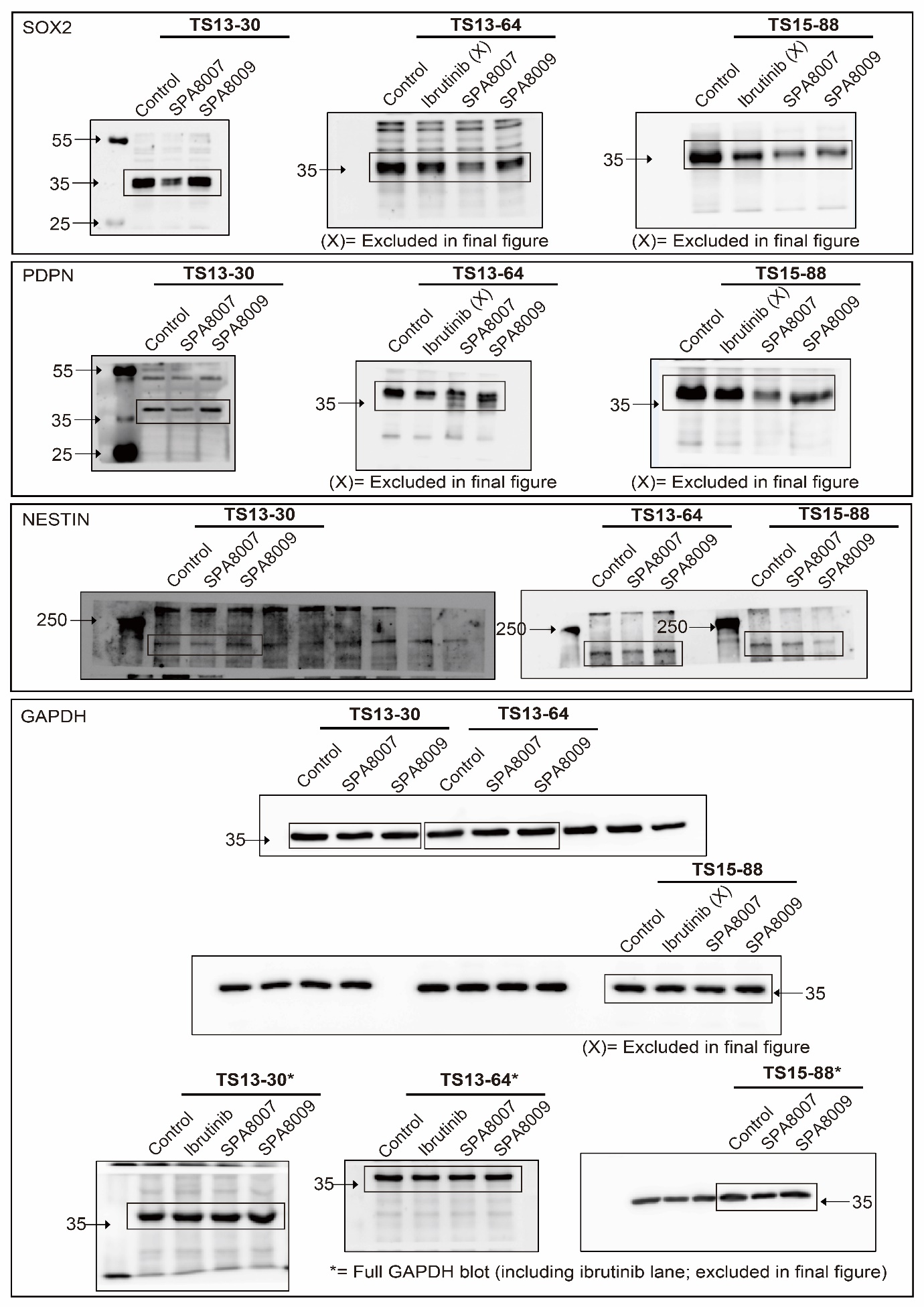
**

**Supplementary Fig. 6**

**
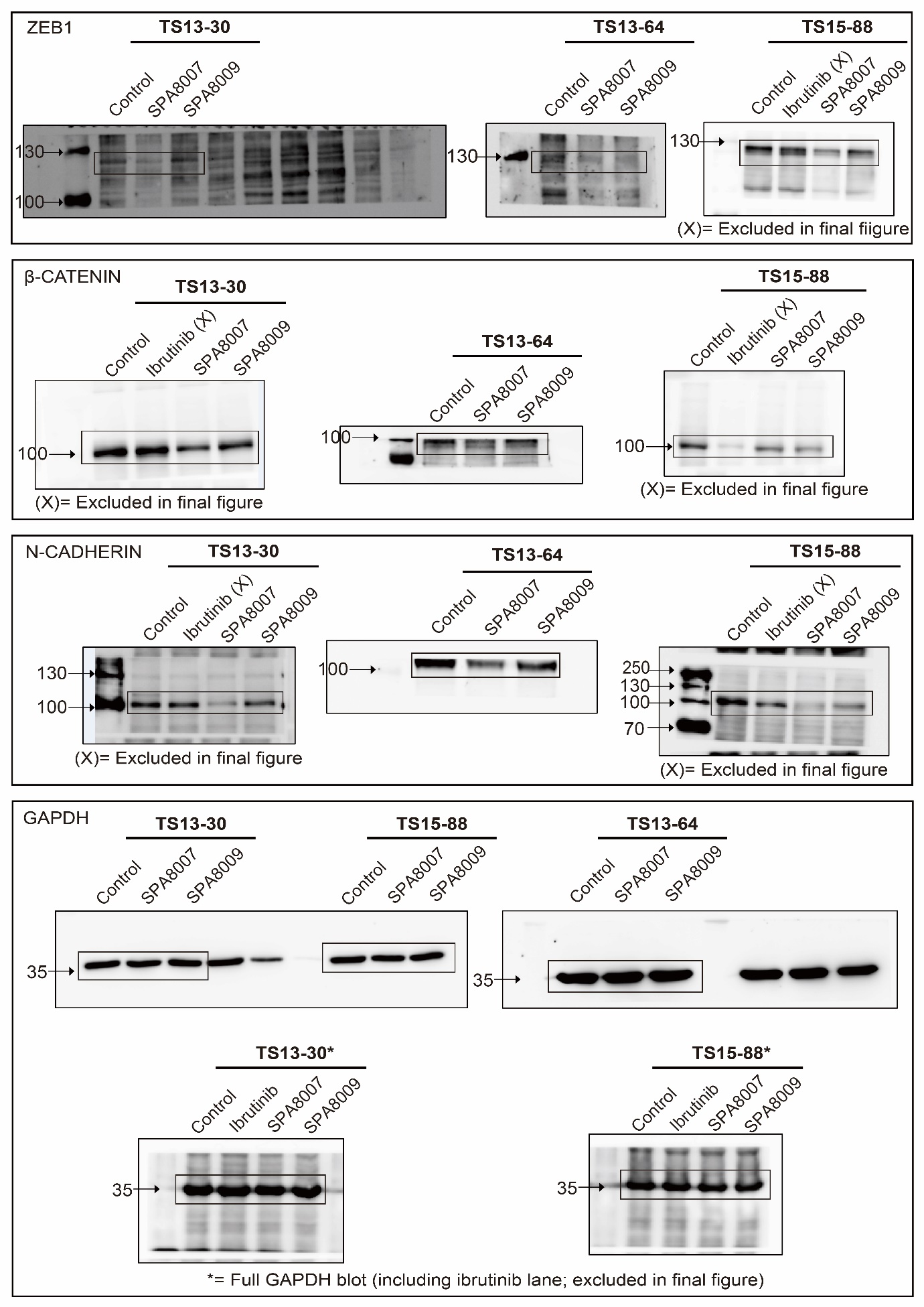
**

**Supplementary Table 1.** Clinical characteristics of tumor free cortex tissues randomly obtained from GBM patients

| Demographics | | Tumor-free cortex  (n= 42) |
| --- | --- | --- |
| **Mean Age** |  | 55.8±14.4 |
| **Sex** | M | 21 |
|  | F | 21 |

Tumor-free cortex (n=42) samples were collected from GBM patients.

**Supplementary Table 2.** Clinico-histopathological characteristics of GBM tumor tissues

| Demographics | | GBM Tumor  (n= 99) |
| --- | --- | --- |
| **Mean Age** |  | 59.7±10.9 |
| **Sex** | M | 56 |
|  | F | 43 |
| **Diagnosis** | GBM | 100% |
| **IDH** | Wild-type | 100% |
| **MGMT** | Methylated | 31.3% |
|  | Unmethylated | 68.7% |
| **1p/19q** | Intact/Intact | 94.9% |
|  | Intact/LOH | 1% |
|  | LOH/Intact | 4% |

GBM tumor (n=99) samples were collected from GBM patients. The table summarizes demographic and clinical parameters, including mean age, sex and relevant histopathological findings, providing insights into the baseline characteristics of the analyzed tissues.

IDH: isocitrate dehydrogenase, MGMT : methylation status of O^6^-DNA methylguanine-methlytransferase promoter, 1p/19q: deletion status of 1p/19q

**Supplementary Table 3**. Histopathological characteristics of GBM tissues and TSs derived from each tissue

| **Sample type** | **Pathology** | **MGMT promotor** | **Co-deletion of 1p/19q** | **Verhaak’s subtype of tissue** | **Prognostic subtype of tissue** |
| --- | --- | --- | --- | --- | --- |
| Tissue 13-30 | IDH wildtype GBM | Unmethylated | Intact/Intact | Mesenchymal | Invasive |
| TS 13-30 | IDH wildtype GBM | Unmethylated | Intact/Intact | Neural | Invasive |
| Tissue 13-64 | IDH wildtype GBM | Unmethylated | Intact/Intact | Classical | Invasive |
| TS 13-64 | IDH wildtype GBM | Unmethylated | Intact/Intact | Classical | Intermediate |
| Tissue 15-88 | IDH wildtype GBM | Unmethylated | Intact/Intact | Mesenchymal | Invasive |
| TS 15-88 | IDH wildtype GBM | Unmethylated | Intact/Intact | Proneural | Intermediate |

Key pathological features, including MGMT promotor, Co-deletion of 1p/19q, corresponding Verhaak’s and prognostic subtypes are summarized providing a comparative analysis between the primary tumor tissues and their in vitro-derived TSs.

**References of Supplementary Table 3**.

Park J, Shim JK, Yoon SJ, Kim SH, Chang JH, Kang SG. Transcriptome profiling-based identification of prognostic subtypes and multi-omics signatures of glioblastoma. *Sci Rep.* 2019; 9(1):10555.

Verhaak RG, Hoadley KA, Purdom E, et al. Integrated genomic analysis identifies clinically relevant subtypes of glioblastoma characterized by abnormalities in PDGFRA, IDH1, EGFR, and NF1. *Cancer Cell.* 2010; 17(1):98-110.

**Supplementary Table 4.** Structure and chemical name of BTK inhibitors.

|  | Compound | Structure | Chemical name |
| --- | --- | --- | --- |
| 1 | SPA1758 |  | (7S)-(+)-Acrylic acid 8,8-dimethyl-2-oxo-6,7-dihydro-2H,8H-pyrano [3,2-g]chromen-7-yl ester |
| 2 | SPA1763 |  | (7S)-(+)-Ethenesulfonic acid 8,8-dimethyl-2-oxo-6,7-dihydro-2H,8H-pyrano [3,2-g]chromen-7-yl ester |
| 3 | SPA8004 |  | Acrylic acid 3-[6-amino-5-(6-benzyloxy-3,4-dihydro-1H-isoquinolin-2-yl)pyridin-3-yloxy]phenyl ester |
| 4 | SPA8007 |  | Acrylic acid 2-[6-amino-5-(6-benzyloxy-3,4-dihydro-1H-isoquinolin-2-yl)pyridin-3-yloxy]phenyl ester |
| 5 | SPA8009 |  | Acrylic acid 3-{6-amino-5-[6-(4-methoxybenzyloxy)-3,4-dihydro-1H-isoquinolin-2-yl]pyridin-3-yloxy}phenyl ester |

Molecular structures and corresponding chemical names of newly synthesized BTK inhibitors are presented offering insights into their structural diversity and potential therapeutic applications.

**Supplementary Table 5.** IC_20_ and IC_50_ of BTK inhibitors in TSs and NHA

| **Cell types** | **Ibrutinib** | | **SPA8007** | | **SPA8009** | |
| --- | --- | --- | --- | --- | --- | --- |
|  | **IC_20_ (µM)** | **IC_50_ (µM)** | **IC_20_ (µM)** | **IC_50_ (µM)** | **IC_20_ (µM)** | **IC_50_ (µM)** |
| **TS13-30** | 3 ±1.37 | 23.98 ±3.09 | 5.2 ±2.29 | 26.42 ±7.50 | 6 ±2.70 | 39.90 ±7.08 |
| **TS13-64** | 19 ±7.69 | 57.71 ±5.83 | 10 ±4.31 | 53.17 ±5.80 | 0.4 ±0.42 | >100 |
| **TS15-88** | 1.2 ±0.15 | 26.29 ±4.40 | 1.3 ±0.42 | >100 | 0.5 ±0.48 | >100 |
| **NHA** | 9.2 ±0.95 | 15.30 ±0.82 | 9.7 ±1.30 | 18.66 ±2.96 | 44 ±4.17 | >100 |

Concentrations of BTK inhibitors required to achieve 20 and 50% inhibition of cell growth.
